# Exercise, Physical Activity, and Sedentary Behavior in the Treatment of Depression: Broadening the Scientific Perspectives and Clinical Opportunities

**DOI:** 10.3389/fpsyt.2016.00036

**Published:** 2016-03-11

**Authors:** Mats Hallgren, Matthew P. Herring, Neville Owen, David Dunstan, Örjan Ekblom, Björg Helgadottir, Olivia Aya Nakitanda, Yvonne Forsell

**Affiliations:** ^1^Division of Epidemiology and Public Health Intervention Research (EPHIR), Department of Public Health Sciences, Karolinska Institutet, Solna, Sweden; ^2^Department of Physical Education and Sport Sciences, University of Limerick, Limerick, Ireland; ^3^Health Research Institute (HRI), University of Limerick, Limerick, Ireland; ^4^Behavioural Epidemiology Laboratory, Baker IDI Heart and Diabetes Institute, Melbourne, VIC, Australia; ^5^Physical Activity Laboratory, Baker IDI Heart and Diabetes Institute, Melbourne, VIC, Australia; ^6^Mary MacKillop Institute for Health Research, Australian Catholic University, Melbourne, VIC, Australia; ^7^The Swedish School of Sport and Health Sciences (GIH), Stockholm, Sweden

**Keywords:** physical activity, exercise, sedentary, depression and anxiety disorders, mental health

Research exploring links between exercise and depression now span several decades, yet several clinically relevant research questions remain unanswered. This opinion article briefly describes the status of selected research issues from the exercise depression literature and offer insights into research areas that are currently lacking. We draw particular attention to the potential of research exploring links between sedentary behavior and depression.

## Benefits of Exercise Interventions in Depression: Evidence from Randomized Controlled Trials

To date, the strongest evidence on the benefits of physical activity in depression comes from randomized controlled trials (RCTs), in which changes in depression severity have been assessed before and after a prescribed exercise intervention (1). Meta-analytic reviews have supported the antidepressant effects of exercise among otherwise healthy adults (2), chronically ill patients (3), and patients with depressive disorders (4). Three recent Cochrane reviews have each concluded that exercise is moderately more effective than a control intervention for reducing depression symptoms (standardized mean difference from 35 trials = −0.62) (4–6). For example, in a multicentre trial involving 2322 patients treated for heart failure, Blumenthal et al. (7) randomized participants to either 12 months of supervised home-based aerobic exercise or usual depression treatment (no planned exercise). Compared with usual care, aerobic exercise resulted in a modest but statistically significant reduction in mean depression scores at both 3- and 12-month follow-up (7). In an often cited efficacy study, Dunn et al. explored dose–response relationships to exercise in 80 adults aged 20–45 years with mild-to-moderate depression (1). Participants were randomized to one of the four supervised exercise treatment groups lasting 12 weeks or a control condition. The findings demonstrated that exercise by itself, in amounts equivalent to consensus public health recommendations, is effective in the treatment of depression.

Recently, physical exercise interventions have also been compared to Internet-delivered treatments for depression. In the largest community-based effectiveness trial of exercise for depression, Hallgren et al. randomized 946 outpatients to one of the three 12-week interventions: prescribed physical exercise, Internet-based cognitive behavioral therapy (ICBT), and usual care by a physician, consisting of brief CBT-focused therapy and antidepressant treatment (8). Supervised aerobic exercise and stretching classes were offered to participants three times per week for 12 weeks. At posttreatment, patients randomized to the exercise intervention reported significantly lower depression severity, compared to usual care, and the improvements were equivalent to those seen in the ICBT group (8).

## Non-Exercise Physical Activity and Depression: Broadening the Perspective

This experimental research provides clinically relevant information about the effects of prescribed *exercise* on mental health outcomes. However, exercise is only one subtype of physical activity, involving planned, repetitive movement, purposefully engaged in to improve fitness and/or health (9). Physical activity has a broader definition and includes all forms of daily movement that result in energy expenditure above resting levels (9). Thus, total physical activity comprises the sum of non-exercise activities (e.g., housework and gardening) and exercise activities (e.g., running and weight training). Importantly, these non-exercise activities contribute a much larger proportion to overall energy expenditure than planned exercise does on a daily basis (10).

Cross-sectional studies have shown that depressed adults are significantly less active than non-depressed adults (11, 12). On average, physically active people have nearly 45% lower odds of experiencing depression symptoms compared to inactive people (13). Several large prospective cohort studies have reported inverse associations between physical activity and depressive symptoms (14, 15), highlighting the important relationship between habitual physical activity levels (as distinct from structured exercise programs) and depression severity in adults. For example, Gudmundsson et al. (16) prospectively followed 676 women over 32 years (1974–2005) with self-report measures of physical activity and depressive symptoms. At baseline, lower levels of activity were associated with higher depression scores and those with decreasing activity levels over a time reported significantly higher depression severity scores at follow-up (16). In another study, Lindwall et al. examined prospective associations between physical activity levels and mental health in 3717 health-care workers (mean age = 46.9 years) across four measurement points spanning 6 years; positive changes in physical activity were associated with positive changes in depression, anxiety, and burnout across time (17).

A notable limitation of previous longitudinal research is that the majority of studies have explored associations of physical activity with depression in non-clinical samples. Indeed, a recent systematic review of 30 prospective studies by Mammen and Faulkner reported that baseline physical activity was negatively associated with the risk of subsequent depression in 25 out of 30 non-clinical, community-based studies (18). This review provides useful information about the relationship between physical activity and the *onset* of depressive illness in otherwise healthy individuals.

However, an equally important question is whether habitual physical activity levels predict the response to depression treatment in individuals who are undergoing treatment. Harris et al. found that higher levels of leisure-time physical activity in 424 depressed adults were associated with lower levels of depression at four assessment points spanning 10 years (19). As in previous studies, however, the authors focused on longitudinal associations generally, rather than the relationship between baseline physical activity levels and the response to depression treatment *per se*.

## Sedentary Behavior and Depression: Too Little Exercise *and* Too Much Sitting

Related to physical activity, sedentary behavior refers to any waking activity characterized by an energy expenditure of ≤1.5 metabolic equivalents and a sitting or reclining posture (20). Common sedentary behaviors include TV viewing, computer use, driving, and reading. High levels of sedentary time have been linked detrimentally with cardiovascular disease, diabetes, and premature mortality (21, 22). Recent studies have also demonstrated associations between sedentary behavior and mental health. In a meta-analysis, Zhai et al. reported that sedentary behavior is significantly associated with an increased risk of depression (23). The pooled risk ratios of depression for sedentary behavior were 1.31 (95% CI = 1.16–1.48) in 13 cross-sectional studies and 1.14 (95% CI = 1.06–1.21) in 11 longitudinal studies.

Liu et al. conducted the first meta-analysis examining associations between “screen time” (a measure of sedentary behavior based on time spent using computers and smartphones) and depression in children and adolescents (24). The analysis examined 12 cross-sectional and 4 longitudinal studies (including 1 cohort study). Results indicated that screen time was associated with depression in a non-linear dose–response manner. The authors concluded that further prospective studies are needed to determine whether mutual causality exists between this key measure of inactivity and depression in young people (24). More generally, Liu et al. (24) review highlighted a need to move down the age spectrum to assess these relationships in adolescents, where it may be particularly important to examine interrelations among the factors known to be important in adult depression.

## Changing Sedentary Behavior in Depression: A Research Priority

Evidence supporting an association between sedentary behavior and depression in adults is growing but currently limited by methodological weaknesses (21). Moreover, studies linking changes in objectively assessed sedentary behaviors with treatment success in depression are currently lacking. Specifically, no studies have compared the effectiveness of a traditional exercise intervention for depression, with an intervention targeting reductions in sedentary behavior. Given the current uncertainty regarding the optimal “dose” of exercise needed to optimize treatment response, an important research question is whether increases in moderate-to-vigorous exercise are necessary to reduce depression or whether equivalent improvements can be achieved through programs that encourage individuals to “move more and sit less.” Current public health guidelines (25) and the bulk of scientific evidence support the former treatment recommendation (4), yet depression interventions targeting sedentary behavior remain absent.

Such research questions could be addressed through RCTs, with the aim of examining the effectiveness of physical activity interventions targeting sedentary behavior, and through prospective cohort studies, where the same individuals are tracked over time using objective assessments of daily activity. RCTs should be adequately powered, should involve an active control group (treatment as usual), and should include long-term follow-up assessments to determine the maintenance of treatment response.

## Exercise, Physical Activity, Sedentary Behavior, and Depression: Clarifying Underlying Mechanisms

The mechanisms underlying relationships of exercise, physical activity, and sedentary behavior with improved depression remain understudied. A myriad of biologically plausible factors have been implicated as putative exercise-related mechanisms, including altered 5-HT, NE, and BDNF (26, 27), increased hippocampal cell proliferation (28, 29), and reduced levels of proinflammatory cytokines (30). The 5-HT system may be particularly important to exercise-induced improvements. Changes in brain 5-HT have been shown following acute and chronic exercise in rats (26). In humans, exercise is thought to alter 5-HT release and metabolism in the brain through increased free fatty acid levels during exercise, which stimulate the potential for enhanced 5-HT synthesis (31). In addition, inflammatory processes may directly and indirectly affect the development and treatment of depression *via* influences on the 5-HT system and interactions with adiposity. Cytokine activation of indoleamine-2,3-dioxygenase leads to relative depletion of tryptophan (TRP) and accumulation of kynurenine (KYN), a neurotoxic by-product shown to induce depression (32). Importantly, recent evidence suggested that skeletal muscle PGC-1α1 induced by exercise training alters KYN metabolism and may protect against stress-induced depression (33). The authors reported that reducing plasma KYN in rats protects the brain from stress-induced changes associated with depression (33).

Higher levels of adiposity may function as both a precursor and a consequence of depression, reflecting a bidirectional relationship that may be driven partly by inflammation (34). For example, the KYN/TRP ratio, which conveys changes in both peripheral blood and brain (35), was found to be significantly higher among overweight/obese adults compared to normal weight controls (36) and has been associated with both depression (37) and obesity (36). Thus, inflammatory markers, particularly in the context of increased adiposity, may contribute to depression, putatively, through interactions with the 5-HT system.

For sedentary behavior, there is evidence – albeit not specifically in the context of depression – identifying relationships with C-reactive protein and other inflammation-related markers (38, 39). However, the independent effects and interaction of inflammatory factors, the 5-HT system, and adiposity have remained unstudied as mechanisms of the antidepressant effects of physical activity and exercise, and clearly warrant future research, particularly as these may be influenced by changing sedentary behaviors.

## Conclusion

In sum, evidence-based treatment for depression continues to expand, but successful treatment and maintenance of treatment response remain limited. Thus, there is a continued need for research into factors that predict successful treatment outcomes. Further research is certainly needed into the effects of planned exercise on depression, including the optimal dose–response and underlying causal mechanisms. However, additional longitudinal studies are also necessary to better understand the complex relationship between habitual physical activity, sedentary behavior, and depression severity. Importantly, these investigations should include children and adolescents. Studies that attempt to assess how changes in levels of activity and inactivity may moderate/mediate the response to empirically supported depression treatments will be particularly relevant. From a public health perspective, it may also be relevant to determine whether those who meet the minimum physical activity levels recommended for general health (40) respond with lower depression severity posttreatment compared to those who do not meet these recommended levels.

With the emerging evidence on the distinct impacts of health outcomes on sedentary behavior – too much sitting as distinct from too little exercise – there is an even broader focus on the overall physical activity spectrum, including the light-intensity physical activities that can displace significant amounts of sedentary time (41). Such broader perspectives on physical activity in the context of depression could be used by clinicians when formulating treatment plans with the goal of maximizing therapeutic benefits, while minimizing the risk of relapse.

Interventions that encourage an overall lifestyle change oriented toward more activity and less sedentary behavior will likely benefit this patient group. However, further research is needed to better understand the plausibly influential roles of both activity and inactivity in this prevalent disorder.

## Author Contributions

Mats Hallgren initiated the article and wrote the first draft. All coauthors provided input into subsequent revisions.

## Conflict of Interest Statement

The authors declare that the research was conducted in the absence of any commercial or financial relationships that could be construed as a potential conflict of interest.

## References

[1] Dunn AL Trivedi MH Kampert JB Clark CG Chambliss HO. Exercise treatment for depression: efficacy and dose response. Am J Prev Med (2005) 28(1):1–8. 10.1016/j.amepre.2004.09.003 15626549

[2] Conn VS. Depressive symptom outcomes of physical activity interventions: meta-analysis findings. Ann Behav Med (2010) 39(20422333):128–38. 10.1007/s12160-010-9172-x 20422333 PMC2870716

[3] Herring MP Puetz TW O’Connor PJ Dishman RK. Effect of exercise training on depressive symptoms among patients with a chronic illness: a systematic review and meta-analysis of randomized controlled trials. Arch Intern Med (2012) 172(22271118):101–11. 10.1001/archinternmed.2011.696 22271118

[4] Cooney GM Dwan K Greig CA Lawlor DA Rimer J Waugh FR Exercise for depression. Cochrane Database Syst Rev (2013) (9):CD004366 10.1002/14651858.CD004366.pub6 24026850 PMC9721454

[5] Mead GE Morley W Campbell P Greig CA McMurdo M Lawlor DA Exercise for depression. Cochrane Database Syst Rev (2008) (4):CD004366 10.1002/14651858.CD004366.pub3 18843656

[6] Rimer J Dwan K Lawlor DA Greig CA McMurdo M Morley W Exercise for depression. Cochrane Database Syst Rev (2012) (7):CD004366 10.1002/14651858.CD004366.pub5 22786489

[7] Blumenthal JA Babyak MA O’Connor C Keteyian S Landzberg J Howlett J Effects of exercise training on depressive symptoms in patients with chronic heart failure: the HF-ACTION randomized trial. JAMA (2012) 308(5):465–74. 10.1001/jama.2012.8720 22851113 PMC3953459

[8] Hallgren M Kraepelien M Ojehagen A Lindefors N Zeebari Z Kaldo V Physical exercise and Internet-based cognitive behavioural therapy in the treatment of depression: randomised controlled trial. Br J Psychiatry (2015) 207(3):227–34. 10.1192/bjp.bp.114.160101 26089305

[9] Caspersen CJ Powell KE Christenson GM. Physical activity, exercise, and physical fitness: definitions and distinctions for health-related research. Public Health Rep (1985) 100(2):126–31. 3920711 PMC1424733

[10] Levine JA. Sick of sitting. Diabetologia (2015) 58(8):1751–8. 10.1007/s00125-015-3624-6 26003325 PMC4519030

[11] Helgadottir B Forsell Y Ekblom O. Physical activity patterns of people affected by depressive and anxiety disorders as measured by accelerometers: a cross-sectional study. PLoS One (2015) 10(1):e0115894. 10.1371/journal.pone.0115894 25585123 PMC4293141

[12] Burton C McKinstry B Szentagotai Tatar A Serrano-Blanco A Pagliari C Wolters M. Activity monitoring in patients with depression: a systematic review. J Affect Disord (2013) 145(1):21–8. 10.1016/j.jad.2012.07.001 22868056

[13] Committee PAGA. Physical Activity Guidelines Advisory Committee Report. Washington, DC: U.S. Department of Health and Human Service (2008). p. 1–683.

[14] Roshanaei-Moghaddam B Katon WJ Russo J. The longitudinal effects of depression on physical activity. Gen Hosp Psychiatry (2009) 31(4):306–15. 10.1016/j.genhosppsych.2009.04.002 19555789

[15] Teychenne M Ball K Salmon J. Physical activity and likelihood of depression in adults: a review. Prev Med (2008) 46(5):397–411. 10.1016/j.ypmed.2008.01.009 18289655

[16] Gudmundsson P Lindwall M Gustafson DR Ostling S Hallstrom T Waern M Longitudinal associations between physical activity and depression scores in Swedish women followed 32 years. Acta Psychiatr Scand (2015) 132(6):451–8. 10.1111/acps.12419 25865488 PMC4600636

[17] Lindwall M Gerber M Jonsdottir IH Borjesson M Ahlborg G The relationships of change in physical activity with change in depression, anxiety, and burnout: a longitudinal study of Swedish healthcare workers. Health Psychol (2014) 33(11):1309–18. 10.1037/a0034402 24245832

[18] Mammen G Faulkner G. Physical activity and the prevention of depression: a systematic review of prospective studies. Am J Prev Med (2013) 45(5):649–57. 10.1016/j.amepre.2013.08.001 24139780

[19] Harris AH Cronkite R Moos R. Physical activity, exercise coping, and depression in a 10-year cohort study of depressed patients. J Affect Disord (2006) 93(1–3):79–85. 10.1016/j.jad.2006.02.013 16545873

[20] Barnes J Behrens TK Benden ME Biddle S Bond D Brassard P Letter to the editor: standardized use of the terms “sedentary” and “sedentary behaviours”. Ment Health Phys Act (2013) 6(1):55–6. 10.1016/j.mhpa.2012.06.001

[21] Teychenne M Costigan SA Parker K. The association between sedentary behaviour and risk of anxiety: a systematic review. BMC Public Health (2015) 15:513. 10.1186/s12889-015-1843-x 26088005 PMC4474345

[22] Dunstan DW Thorp AA Healy GN. Prolonged sitting: is it a distinct coronary heart disease risk factor? Curr Opin Cardiol (2011) 26(5):412–9. 10.1097/HCO.0b013e3283496605 21785350

[23] Zhai L Zhang Y Zhang D Sedentary behaviour and the risk of depression: a meta-analysis. Br J Sports Med (2015) 49(11):705–9. 10.1136/bjsports-2014-093613 25183627

[24] Liu M Wu L Yao S. Dose-response association of screen time-based sedentary behaviour in children and adolescents and depression: a meta-analysis of observational studies. Br J Sports Med (2015). 10.1136/bjsports-2015-095084 26552416 PMC4977203

[25] Sundberg CJ Jansson A Edling C Wadman M Physical Activity in the Prevention and Treatment of Disease. Östersund: Professional Associations for Physical Activity, Produced in Cooperation with Swedish National Institute of Public Health (2010). p. 1–630.

[26] Dishman RK. Brain monoamines, exercise, and behavioral stress: animal models. Med Sci Sports Exerc (1997) 29(1):63–74. 10.1097/00005768-199701000-00010 9000157

[27] Laske C Banschbach S Stransky E Bosch S Straten G Machann J Exercise-induced normalization of decreased BDNF serum concentration in elderly women with remitted major depression. Int J Neuropsychopharmacol (2010) 13(5):595–602. 10.1017/S1461145709991234 20067661

[28] Dunn AL Jewell JS. The effect of exercise on mental health. Curr Sports Med Rep (2010) 9(4):202–7. 10.1249/JSR.0b013e3181e7d9af 20622537

[29] Bjornebekk A Mathe AA Brene S. The antidepressant effect of running is associated with increased hippocampal cell proliferation. Int J Neuropsychopharmacol (2005) 8(3):357–68. 10.1017/S1461145705005122 15769301

[30] Eyre HA Papps E Baune BT. Treating depression and depression-like behavior with physical activity: an immune perspective. Front Psychiatry (2013) 4:3. 10.3389/fpsyt.2013.00003 23382717 PMC3562851

[31] Chaouloff F. Effects of acute physical exercise on central serotonergic systems. Med Sci Sports Exerc (1997) 29(1):58–62. 10.1097/00005768-199701000-00009 9000156

[32] Miller AH Maletic V Raison CL. Inflammation and its discontents: the role of cytokines in the pathophysiology of major depression. Biol Psychiatry (2009) 65(19150053):732–41. 10.1016/j.biopsych.2008.11.029 19150053 PMC2680424

[33] Agudelo LZ Femenia T Orhan F Porsmyr-Palmertz M Goiny M Martinez-Redondo V Skeletal muscle PGC-1alpha1 modulates kynurenine metabolism and mediates resilience to stress-induced depression. Cell (2014) 159(1):33–45. 10.1016/j.cell.2014.07.051 25259918

[34] Luppino FS de Wit LM Bouvy PF Stijnen T Cuijpers P Penninx BWJH Overweight, obesity, and depression: a systematic review and meta-analysis of longitudinal studies. Arch Gen Psychiatry (2010) 67(20194822):220–9. 10.1001/archgenpsychiatry.2010.2 20194822

[35] Raison CL Dantzer R Kelley KW Lawson MA Woolwine BJ Vogt G CSF concentrations of brain tryptophan and kynurenines during immune stimulation with IFN-α: relationship to CNS immune responses and depression. Mol Psychiatry (2009) 15(4):393–403. 10.1038/mp.2009.116 19918244 PMC2844942

[36] Mangge H Summers KL Meinitzer A Zelzer S Almer G Prassl R Obesity-related dysregulation of the tryptophan-kynurenine metabolism: role of age and parameters of the metabolic syndrome. Obesity (2014) 22(1):195–201. 10.1002/oby.20491 23625535

[37] Gabbay V Klein RG Katz Y Mendoza S Guttman LE Alonso CM The possible role of the kynurenine pathway in adolescent depression with melancholic features. J Child Psychol Psychiatry (2010) 51(8):935–43. 10.1111/j.1469-7610.2010.02245.x 20406333 PMC3711227

[38] Healy GN Matthews CE Dunstan DW Winkler EA Owen N. Sedentary time and cardio-metabolic biomarkers in US adults: NHANES 2003-06. Eur Heart J (2011) 32(5):590–7. 10.1093/eurheartj/ehq451 21224291 PMC3634159

[39] Howard BJ Balkau B Thorp AA Magliano DJ Shaw JE Owen N Associations of overall sitting time and TV viewing time with fibrinogen and C reactive protein: the AusDiab study. Br J Sports Med (2015) 49(4):255–8. 10.1136/bjsports-2013-093014 24550208

[40] Garber CE Blissmer B Deschenes MR Franklin BA Lamonte MJ Lee IM American College of Sports Medicine position stand. Quantity and quality of exercise for developing and maintaining cardiorespiratory, musculoskeletal, and neuromotor fitness in apparently healthy adults: guidance for prescribing exercise. Med Sci Sports Exerc (2011) 43(7):1334–59. 10.1249/MSS.0b013e318213fefb 21694556

[41] Tremblay MS Colley RC Saunders TJ Healy GN Owen N. Physiological and health implications of a sedentary lifestyle. Appl Physiol Nutr Metab (2010) 35(6):725–40. 10.1139/H10-079 21164543

